# The Characteristics of Demineralized Dentin Material Sponge as Guided Bone Regeneration Based on the FTIR and SEM-EDX Tests

**DOI:** 10.1055/s-0042-1743147

**Published:** 2022-03-13

**Authors:** Indra Mulyawan, Coen Pramono Danudiningrat, Pratiwi Soesilawati, Aulanni'am Aulanni'am, Anita Yuliati, Heri Suroto, Taufan Bramantoro, Andra Rizqiawan, Seong-Yong Moon

**Affiliations:** 1 Departement of Oral and Maxillofacial Surgery, Faculty of Dentistry, Universitas Airlangga, Surabaya, Indonesia; 2 Departement of Biology Oral, Faculty of Dentistry, Universitas Airlangga, Surabaya, Indonesia; 3 Departement of Chemistry, Faculty of Science, Universitas Brawijaya, Malang, Indonesia; 4 Departement of Dental Material, Faculty of Dentistry, Universitas Airlangga, Surabaya, Indonesia; 5 Departement of Orthopaedic and Traumatology Surgery, Faculty of Medicine, Universitas Airlangga, Surabaya, Indonesia; 6 Cell and Tissue Bank, Regenerative Medicine, Dr. Soetomo General Academic Hospital, Surabaya, Indonesia; 7 Departement of Dental Public Health, Faculty of Dentistry, Universitas Airlangga, Surabaya, Indonesia; 8 Departement of Oral and Maxillofacial Surgery, Faculty of Dentistry, Chosun University, Gwangju, South Korea

**Keywords:** tissue engineering, guided bone regeneration, demineralized dentin, Fourier transform infrared, SEM-EDX

## Abstract

**Objective** The objective of this study was to determine the characteristics of demineralized dentin material sponge (DDMS).

**Material and Methods** An observational study was conducted on DDMS and BPCM. Fourier transform infrared (FTIR) test was performed to determine the characterizations of the materials. Scanning electron microscope-electron dispersive X-ray spectroscopy (SEM-EDX) test was performed to observe the elements contained in the materials.

**Results** The infrared spectrum of the DDMS and BPCM functional groups showed the same pattern in each variation, and no significant differences were found. According to SEM analysis, the cavities that make up the membrane were spotted on the surface. Besides, according to the SEM-EDX analysis, DDMS contained chlorine, carbon, and calcium, while BPCM contained carbon, oxygen, and sulfur.

**Conclusion** DDMS has the potential to be a biomaterial for bone tissue engineering in terms of the characteristics. DDMS had a structure that almost resembles BPCM as seen from the results of the FTIR graph between DDMS and BPCM. The morphological structure of the two materials in the SEM test appeared to have porosity with various sizes.

## Introduction

Head and facial bone defects occur when the bones in the cranial and facial areas lose their tissues. 1 The most common observation of insufficient quantity of bone in dentistry is following tooth loss, where rapid resorption of alveolar bone occurs due to the absence of intraosseous stimulation that would typically occur via the periodontal ligament fibers. These defects can lead to reduced cranial bone function and anatomic changes. 2 3 Tissue engineering has currently become an alternative procedure for bone repair. While engineered tissue can be made from the combination of stem cells, growth factors, and scaffolds, one of the techniques that can be used to produce engineered tissue is by forming bone cells through the guided bone regeneration (GBR) method. 4 5

The GBR as a physical barrier device consists of two membrane types, i.e., bioabsorbable and non-resorbable which function to create a space around the defect as a guide for the bone regeneration and reconstruction process as well as prevent the invasion of fibrous connective tissue into the bone defect. The GBR collagen membrane materials include bovine pericardium collagen membrane (BPCM) which is proven efficient as GBR. An *in vivo* test showed that the porous structure of BPCM allows cell migration and bone growth into the pericardial matrix. However, this procedure requires expensive technology and is costly since bioabsorbable and non-resorbable membrane products are not cheap. Therefore, alternatives for qualified and more affordable GBR collagen membranes are needed. 6

Derived from bovine dentin, demineralized dentin matrix (DDM) is a synthetic material used as a substitute for human bone. In recent years, DDM has been widely used in dental research due to its more uniform composition similar to humans. Besides, the popular use of DDM is also influenced by the fact that it has high quality and can be easily obtained in large quantities. Bovine dentin has a composition similar to human dentin which consists of 70% inorganic matter, 20% organic matter, and 10% water. 7 8 The dentin microstructure consists of a network of collagen fibers and contains various growth factors, such as insulin-2 growth factor, bone morphogenetic protein (BMP), tumor-β growth factor, platelet-derived growth factor, and fibroblast growth factor. 9

DDM can be an important source of bone regeneration materials as well as microporous ones due to its organic and inorganic components. DDM is known to be more inductive than mineralized dentin, mainly due to the release of type-I collagen (COL-I) matrix growth factors such as BMP as well as osteocalcin, osteonectin, and dentin phosphoproteins which are known to be involved in bone mineralization. DDM has higher osteoinductive efficiency and simultaneously induces bone growth. DDM can be considered as a complex COL-I and growth factor which has lost the bound mineral crystals, which are released from the matrix. It has significant osteoinductive and osteoconductive biological effects. Autologous and xenogenous DDMs have been used to treat bone injury and bone damage. Therefore, DDM is considered competent enough as a bone regeneration and replacement material. 10 11

Based on the above explanation, this study aimed to determine the characteristics of demineralized dentin material sponge (DDMS). Fourier transform infrared (FTIR) test was performed to determine the characteristics and the components contained in the materials through functional groups. Moreover, scanning electron microscope-electron dispersive X-ray spectroscopy (SEM-EDX) test was performed to assess functional groups contained in the materials.

## Material and Methods

An observational study was conducted on DDMS and BPCM. DDMS was produced at the Network Bank of District General Hospital of Dr. Soetomo Surabaya, while BPCM/Jason Membrane was produced at Botiss Dental Biomaterial GmbH.

### Preparation of DDMS

Bovine teeth were cleaned with peroxidase 3% for 1 week. The crowns and roots were subsequently separated by means of a bone cutter and rongeur forceps. Powder was produced by processing the teeth comprised of dentin and cementum tissue with a bone miller. The resulting products were filtered until the desired amount was obtained. The demineralization process was performed using a bone mineral removal method involving immersion in 1% hydrochloric acid (Merck, NJ, United States) for a day and thorough rinsing before being dried. Prior to storage, the protein content of the tooth was frozen (freeze-dried = lyophilization). The sponge samples were freeze-dried using a tray freeze-dryer under −80°C.

### FTIR Test

DDMS and BPCM were characterized using the FTIR instrument to identify functional groups and provide structural information. The range of wavenumbers used is 4,000 to 400 cm ^−1^ . An FTIR test kit (Thermo Scientific, Nicolet, iS10) was connected to the monitor, and the OMNIC program was opened. Each sample was then calibrated by selecting the “collect sample” button in the program. As the program displayed the graph, the sample was then placed in the center of the FTIR test instrument holder and pressed. Next, the OMNIC program was opened, and its wavelength was set at 4,000 to 400 cm ^−1^ . The results of the FTIR test were described in a graph.

### SEM-EDX Test

DDMS and BPCM were characterized using the SEM-EDX instrument to identify the largest porosity. SEM (FEI, Inspect-S50) was connected to EDX spectroscopy. The SEM holder was then coated with double-sided carbon tape. One side was attached to the SEM holder, and the other side was attached to the sample. Since DDMS and BPCM used as the samples in this study were non-conducting materials, they had to go through the coating process first. Coating was done by inserting the samples and the holder on the sputter coater and vacuuming for 30 minutes. After the process of vacuum was completed, plasma coating was performed for 3 seconds using Au and Pb. The coated samples were then put into the SEM holder. The coated samples were vacuumed again for 5 minutes. Images were taken at 500x, 2,000x, and 5,000x magnification to measure the pores with the largest porosity. The measurement process was done by drawing a line on the selected pores. The pores were obtained according to the required number. Then, the EDX program was run, and the test results were explained in graphs and tables of the element percentage.

## Results

**Fig. 1:**
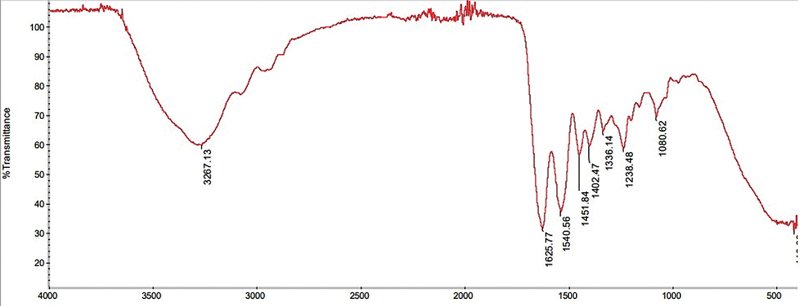
The FTIR DDMS graph.

**Fig. 2:**
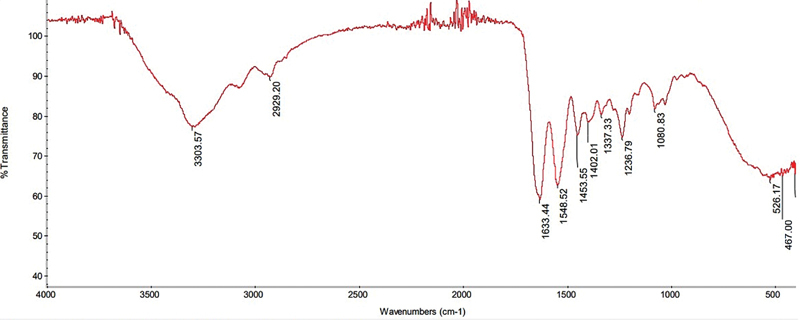
The FTIR BPCM graph.

The FTIR spectrum pattern is presented in Figs. 1 and 2 . The FTIR spectrum pattern of DDMS and BPCM showed peaks that indicated the functional groups of the compound for DDMS and BPCM. Based on the IR spectrum, the DDMS and BPCM functional groups showed the same pattern in each variation, and no significant differences were found.

**Fig. 3:**
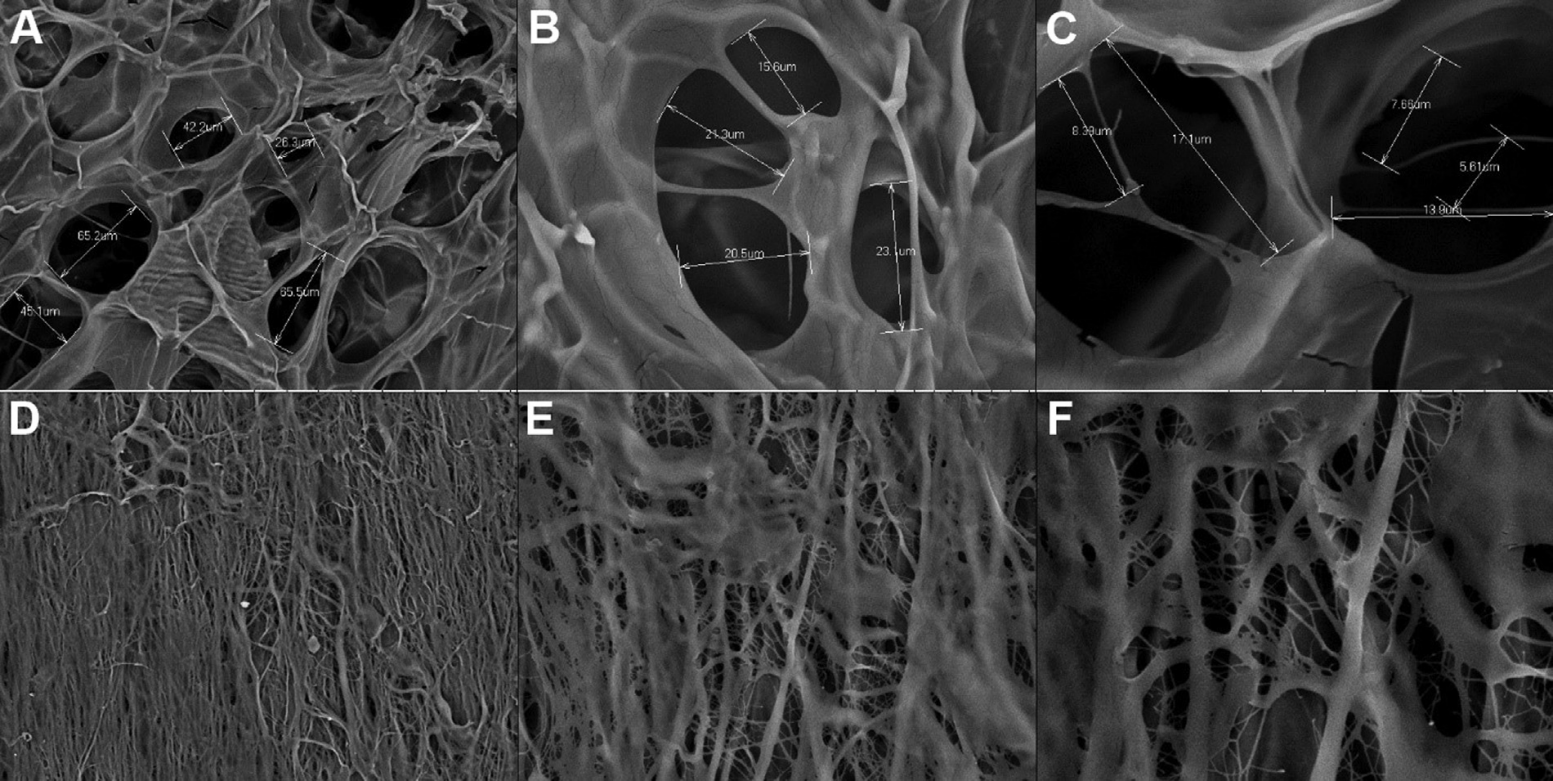
The porosity of DDMS and BPCM. ( **A** ) DDMS with 500x magnification. ( **B** ) DDMS with 2,000x magnification. ( **C** ) DDMS with 5,000x magnification. ( **D** ) BPCM with 500x magnification. ( **E** ) BPCM with 2,000x magnification. ( **F** ) BPCM with 5,000x magnification.

Based on the SEM test, it was found that the membrane was composed of the cavities that appeared on the surface of the samples. The size of the cavities ranged from small to large as shown in Fig. 3 .

**Fig. 4:**
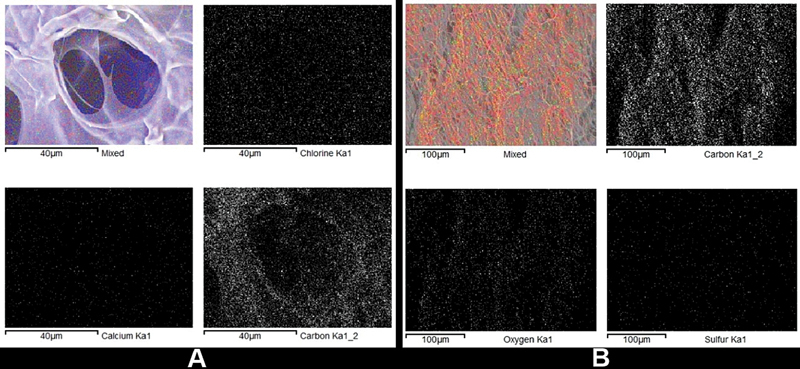
Analysis results of SEM-EDX on ( **A** ) DDMS and ( **B** ) BPCM.

It was then found that the elements contained in DDMS were chlorine, carbon, and calcium, while in BPCM were carbon, oxygen, and sulfur. The results of the SEM-EDX analysis of DDMS and BPCM membranes can be overviewed in Fig. 4 and Tables 1 and 2 .

**Table 1:** Analysis table results of SEM-EDX on DDMS

Element	Weight %	Weight % σ	Atomic %
Carbon	57.923	0.412	67.909
Oxygen	32.705	0.390	28.786
Sodium	2.158	0.060	1.322
Sulfur	0.309	0.033	0.136
Chlorine	2.959	0.060	1.175
Calcium	0.325	0.033	0.114
Zirconium	3.621	0.120	0.559

Abbreviations: DDMS, demineralized dentin material sponge; SEM-EDX, scanning electron microscope-electron dispersive X-ray spectroscopy.

**Table 2:** Analysis table results of SEM-EDX on BPCM

Element	Weight %	Weight % σ	Atomic %
Carbon	67.218	0.516	75.459
Oxygen	28.188	0.513	23.757
Sulfur	0.385	0.050	0.162
Zirconium	4.208	0.181	0.622

Abbreviations: BPCM, bovine pericardium collagen membrane; SEM-EDX, scanning electron microscope-electron dispersive X-ray spectroscopy.

## Discussion

Bone and dentin are mineralized tissues with a similar chemical composition. Its composition consists of 18% collagen, 2% non-collagen protein (NCP), 70% hydroxyapatite (HA) and 10% body fluids (percentage indicates weight /volume). About 90% of the organic material in dentin consists of COL-I fibers. 12 The remaining organic components consist of NCP, phosphorylated, and nonphosphorylated proteins. 13 Pang et al 14 conducted a randomized controlled clinical trial of AutoBT (autogenous human DDM, Korea Tooth Bank, Seoul, Korea) to compare bovine bone with inorganic bone for socket extraction augmentation. Vertical dimensional augmentation showed that it was as effective as augmentation using bovine inorganic bone both groups demonstrated favorable wound healing, implant stability, and histologically confirmed new bone formation. The results of this study indicate that autogenous bone graft is a viable choice for alveolar bone augmentation after tooth extraction. 12 *In vivo* studies have shown that DDM is a more effective bone-strengthening matrix than calcified dentin matrix (CDM) because CDM granules induce the formation of only a small amount of bone after a latency period of 8 to 12 weeks. The inductive nature of CDM may be related to the inhibition of BMP release by apatite crystals. 13

The FTIR spectrum indicated several DDMS and BPCM wavenumbers as shown in Figs. 1 and 2 . The DDMS wavenumber 3,267.13 cm ^−1^ indicated the presence of phenol group (O-H), 1,540.56 cm ^−1^ indicated the aromatic ring group (C = C), and 1,080.62 cm ^−1^ indicated the amino group (C-O). The FTIR test on demineralized dentin slices reached the peak at 3,312 cm ^−1^ that also indicated the –OH bond strain vibration. Meanwhile, the peak 1,647 cm ^−1^ indicated the amide band I, 1,550 cm ^−1^ indicated the amide band II, and 1,239 cm ^−1^ indicated the amide band III. 15 This figure accords with the FTIR spectrum pattern of DDMS and, thus, suggested that the dentin slices had been demineralized. A similar pattern was also observed in BPCM with a wavenumber of 2,929.2 cm ^−1^ , indicating the alkane group (C-H), 1,548.52 cm ^−1^ , indicating the aromatic ring group (C = C), and 1,080.83 cm ^−1^ , indicating the C-O amino group.

Based on the FTIR test, it was found that both DDMS and BPCM showed almost the same functional groups. Besides, the functional groups in the two materials also resembled those contained in human bone. It is understood that human bone is a biocomposite (an inorganic phase immersed in the organic phase of collagen). The inorganic phase of bone consists of bone apatite (PO43-, OH, and CO32- groups), while the organic phase of bone consists of collagen protein (C = O, CN, COC, and OH groups). These clusters can also be seen in the FTIR spectrum of DDMS and BPCM. 16 17 18

Based on the projection of the SEM test, it was found that the two samples showed many pores with irregular shapes which were evenly distributed over the entire surface. The irregularity of the pore shape could have resulted from freeze-drying, the manufacturing method which tends to produce irregular and scattered pores. 19 20 ECM protein adsorption on the scaffold was influenced by the surface properties and porosity of the scaffold. Good adsorption of ECM protein can increase the strength of cell attachment to the scaffold. An *in vitro* test suggested that osteoblast cells could adhere, proliferate, and differentiate well on the surface of porous materials. 21

The SEM-EDX analysis suggested that there was calcium contained in DDMS but not in BPCM. The main component of dental inorganic material contained several types of calcium phosphate including HA. Moreover, the levels of calcium, phosphorus, and oxygen in the dental inorganic material were usually low. Meanwhile, there was also a very low HA found in the dental inorganic material. Carbon was used as a marker for organic components, and the organic part is mainly carbon-based molecules. The carbon found in the EDX analysis is considered organic. 22

This study found that dentin could be used as a material for bone regeneration. DDM performed better for bone induction than calcified dentin at 4 weeks after implantation. 11 23 DDM is a collagen agent with less antigenicity for releasing growth factors such as bone morphogenic proteins (BMPs), clinically applied as a bone filling agent in the maxillofacial region. Common production methods for DDM involve crushing and demineralizing dentin, including cementum and removing enamel. DDM transplant therapy developed by several studies by Urist and other researchers showed that demineralized bone and dentin transplantation trigger bone formation. 14 Demineralization of dentin increases the bio-availability of matrix-associated non-collagenous proteins, making these grafts osteoinductive. Non-collagenous dentin proteins such as osteocalcin, osteonectin, phosphoprotein, and sialoprotein are also the same elements found in calcified dentin. 24

Demineralization of dentine can increase the bioavailability of matrix-associated non-collagenous proteins such as dentin sialophosphoprotein, dentin phosphoprotein, osteocalcin, osteonectin, and BMP. These proteins can promote a new bone formation. 25 26 The purpose of demineralization is not to completely remove minerals from the dentin but rather to modify the dentin surfaces facing the surrounding tissue (periosteum and bone surfaces). 22

## Conclusion

DDMS has the potential to be a biomaterial for bone tissue engineering in terms of characteristics. DDMS has almost the same structure as BPCM. This could be seen from the comparison of their spectrum as shown in the FTIR test. Both DDMS and BPCM had almost the same functional groups that also resembled those of human bone. Meanwhile, based on the SEM test, the morphological structure of DDMS and BPCM appeared to have porosity with various sizes. According to the EDX test, DDMS had more organic content than BPCM because it went through a demineralization process.
